# Integrating trans-omics, cellular experiments and clinical validation to identify ILF2 as a diagnostic serum biomarker and therapeutic target in gastric cancer

**DOI:** 10.1186/s12885-024-12175-z

**Published:** 2024-04-15

**Authors:** Shao-Song Liu, Qin-Si Wan, Cong Lv, Jin-Ke Wang, Song Jiang, Dan Cai, Mao-Sheng Liu, Ting Wang, Kun-He Zhang

**Affiliations:** Department of Gastroenterology, The First Affiliated Hospital, Jiangxi Medical College, Nanchang University; Jiangxi Institute of Gastroenterology & Hepatology, Nanchang, China, No 17, Yongwai Zheng Street, 330006 Nanchang, China

**Keywords:** Gastric cancer, Diagnosis, Serum biomarker, ILF2, Trans-omics analysis, Immune infiltration, Immunotherapy, Chemotherapy

## Abstract

**Background:**

Gastric cancer (GC) lacks serum biomarkers with clinical diagnostic value. Multi-omics analysis is an important approach to discovering cancer biomarkers. This study aimed to identify and validate serum biomarkers for GC diagnosis by cross-analysis of proteomics and transcriptomics datasets.

**Methods:**

A cross-omics analysis was performed to identify overlapping differentially expressed genes (DEGs) between our previous aptamer-based GC serum proteomics dataset and the GC tissue RNA-Seq dataset in The Cancer Genome Atlas (TCGA) database, followed by lasso regression and random forest analysis to select key overlapping DEGs as candidate biomarkers for GC. The mRNA levels and diagnostic performance of these candidate biomarkers were analyzed in the original and independent GC datasets to select valuable candidate biomarkers. The valuable candidate biomarkers were subjected to bioinformatics analysis to select those closely associated with the biological behaviors of GC as potential biomarkers. The clinical diagnostic value of the potential biomarkers was validated using serum samples, and their expression levels and functions in GC cells were validated using in vitro cell experiments.

**Results:**

Four candidate biomarkers (ILF2, PGM2L1, CHD7, and JCHAIN) were selected. Their mRNA levels differed significantly between tumor and normal tissues and showed different diagnostic performances for GC, with areas under the receiver operating characteristic curve (AUROCs) of 0.629–0.950 in the TCGA dataset and 0.736–0.840 in the Gene Expression Omnibus (GEO) dataset. In the bioinformatics analysis, only ILF2 (interleukin enhancer-binding factor 2) gene levels were associated with immune cell infiltration, some checkpoint gene expression, chemotherapy sensitivity, and immunotherapy response. Serum levels of ILF2 were higher in GC patients than in controls, with an AUROC of 0.944 for the diagnosis of GC, and it was also detected in the supernatants of GC cells. Knockdown of ILF2 by siRNA significantly reduced the proliferation and colony formation of GC cells. Overexpression of ILF2 significantly promotes the proliferation and colony formation of gastric cancer cells.

**Conclusions:**

Trans-omics analysis of proteomics and transcriptomics is an efficient approach for discovering serum biomarkers, and ILF2 is a potential diagnostic biomarker and therapeutic target of gastric cancer.

**Supplementary Information:**

The online version contains supplementary material available at 10.1186/s12885-024-12175-z.

## Introduction

Gastric cancer (GC) is the fifth most common cancer and the fourth leading cause of cancer-related deaths globally, posing a major threat to public health [1].. Many GC patients present with nonspecific symptoms, resulting in diagnosis at an advanced stage and, consequently, an unfavorable prognosis.

GC lacks useful serum biomarkers for clinical diagnosis. Traditional serum biomarkers, such as carcinoembryonic antigen (CEA), carbohydrate antigen 19 − 9 (CA19-9), carbohydrate antigen 72 − 4 (CA72-4), and carbohydrate antigen 125 (CA125), provide minimal diagnostic value for GC [2, 3]. Over the past few decades, several circulating biomarkers that indicate the presence of GC have been documented, including gastrin-17 (G-17), pepsinogens (PGs), anti-Helicobacter pylori IgG antibodies [4], trefoil factor 3 (TFF3) [5], and metabolic markers such as 3-hydroxypropionic acid [6]. In recent years, this category has been expanded to include circulating tumor cells (CTCs) [7], circulating tumor DNA (ctDNA) [8], and non-coding RNAs (miRNA, lncRNA, and circRNA) [9, 10]. However, the utilization of these biomarkers in clinical settings has been hindered by inadequate diagnostic efficacy and technical complexity. Therefore, it is crucial to discover new serum biomarkers for GC diagnosis.

The combined analysis of proteomics and transcriptomics, a multi-omics approach, holds significant potential in discovering cancer biomarkers [11, 12]. Proteins, as the primary executors of cellular functions, directly reflect changes in cellular states and biological processes, while gene transcription levels offer insights into the potential regulatory mechanisms underlying protein expression. By integrating data from these two layers, researchers can more precisely identify differentially expressed proteins and genes associated with tumorigenesis. This approach facilitates the discovery of novel cancer biomarkers, providing a more solid foundation for early diagnosis, prognostic assessment, and treatment selection [13, 14].

In recent years, cancer immunotherapy has emerged as an innovative and promising approach to cancer treatment and has made remarkable advancements [15]. The integration of bioinformatics analysis with publicly available databases has been particularly effective in identifying potential targets for immunotherapy [16, 17]. The application of multi-omics methods, which combine data from various biological levels, has facilitated the discovery of novel targets that can significantly improve the survival rates of GC patients. The exploration of immunotherapy targets through bioinformatics and multi-omics approaches holds great promise for improving outcomes in GC and other cancers.

In our previous aptamer-based proteomics study on GC serum, we identified 236 proteins that were differentially expressed in GC serum compared to healthy controls. In the current study, we aimed to further refine this set of proteins and identify those with diagnostic potential for GC. We selected the differentially expressed GC serum proteins that were differentially expressed at the transcriptional level in The Cancer Genome Atlas-Stomach Adenocarcinoma (TCGA-STAD) dataset, followed by lasso regression and random forest analysis to identify key biomarkers. The key biomarkers were independently evaluated for their diagnostic value in GC and bioinformatically analyzed for their associations with various biological behaviors of GC to identify potential serum biomarkers, and the latter were finally validated for their clinical diagnostic value in serum samples and their biological functions in vitro cellular experiments.

## Methods

### Data collection and processing

The differentially expressed serum proteins in our previous aptamer-based GC serum proteomics dataset were identified and their corresponding genes were searched in the UniProt database (https://www.uniprot.org/). In the TCGA online database (https://portal.gdc.cancer.gov/), mRNA expression data (in transcripts per million) and clinical information were extracted from the STAD dataset (375 tumor tissue samples and 32 normal gastric tissue samples). Differentially expressed genes (DEGs) were identified using the filter criteria of fold change > 1.3 and false discovery rate < 0.05. A cross-analysis between the DEGs in the TCGA-STAD dataset and the genes corresponding to the differentially expressed serum proteins in the GC proteomics dataset was performed to determine overlapping DEGs.

### Trans-omics analysis to identify candidate biomarkers of gastric cancer

Least absolute shrinkage and selection operator (LASSO) regression and random forest (RF) algorithms were used to identify key genes from the overlapping DEGs, and the key genes that overlapped in the results of the two algorithms were designated as candidate biomarkers of GC. The optimal α (λ) value (a parameter that controls feature selection) for LASSO regression was selected by 10-fold cross-validation [18]. For random forest, the decision tree with the lowest out-of-bag (OOB) error rate was selected to evaluate feature importance using the mean decrease gini parameter, and features with the parameter > 2 were selected as key genes [19].

### Internal and external validation

The candidate biomarkers were validated internally using the TCGA-STAD dataset and externally using the Gene Expression Omnibus (GEO) datasets at the mRNA level. Three GEO Series (GSE) datasets were downloaded for external validation: GSE27342 (normal samples: 80; tumor samples: 80), GSE54129 (normal samples: 21; tumor samples: 111), and GSE66229 (normal samples: 100; tumor samples: 300). Batch correction was performed on the three datasets using the “affy” package in the R software. Following dimension reduction through principal component analysis (PCA), a merged GSE dataset containing 201 normal samples and 491 tumor samples was created for external validation.

### Evaluation of the diagnostic value of candidate biomarkers for GC

The diagnostic performance of the mRNA levels of the potential biomarkers for GC was assessed in both the TCGA-STAD dataset and the merged GSE dataset using the area under the receiver operating characteristic curve (AUROC) as a metric.

### Potential biomarkers expression and tumor immune infiltration

To examine the differences in immune cell infiltration between the high and low mRNA levels of the potential biomarkers in GC tissues, we employed Cell-type Identification by Estimating Relative Subsets of RNA Transcripts (CIBERSORT) [20]. This tool analyzes immune cell infiltration by assessing gene expression data [21]. We compared the transcriptional levels of genes associated with immune checkpoints between the high and low mRNA level groups of these potential biomarkers [22, 23], including TIGIT (T-cell immunoglobulin and ITIM domain), SIGLEC15 (Sialic acid-binding immunoglobulin-like lectin 15), HAVCR2 (T-cell immunoglobulin and mucin domain-containing protein 3), CD274 (Cluster of Differentiation 274, also known as PD-L1), PDCD1 (Programmed cell death protein 1, also known as PD-1), LAG3 (Lymphocyte-activation gene 3), CTLA4 (Cytotoxic T-lymphocyte-associated antigen 4), and PDCD1LG2 (Programmed cell death 1 ligand 2, also known as PD-L2).

### Sensitivity to chemotherapy and immunotherapy outcomes based on potential biomarkers expression levels

Data from The Cancer Immunome Atlas (TCIA) database (https://www.tcia.at/home) [24, 25] were used to predict the impact of mRNA levels of potential biomarkers on the response to immunotherapy with CTLA4 and PD-1 inhibitors. The Cancer Drug Sensitivity Genomics (GDSC) database (www.cancerRxgene.org) [26, 27] was used to investigate variations in chemotherapy sensitivity based on high and low mRNA levels of these potential biomarkers.

### ELISA validation

To confirm the diagnostic significance of potential biomarkers, we obtained serum samples from 30 individuals diagnosed with GC and 22 healthy individuals as controls. Serum levels of ILF2 were measured using a commercially available enzyme-linked immunosorbent assay (ELISA) kit (SAB, USA) according to the manufacturer’s instructions. Briefly, 100 μL of serum diluted 1:50 was incubated with capture antibodies at 37 °C for 2 h. After removal of the liquid, 100 μL of biotinylated antibody was added and incubated for one hour. After washing three times, streptavidin-HRP conjugate was added and incubated for 1 h. The liquid was then discarded, the wells were washed five times, and tetramethyl benzidine (TMB) substrate was added and incubated for 15 min in the dark. After stopping the reaction, the optical density (OD) values were measured at 450 nm using a JS-THERMO Varioskan Flash (Thermo Fisher Scientific, USA). A standard curve was simultaneously generated and used to determine serum concentrations of the potential biomarkers. The AUROC was used to evaluate the diagnostic value of the potential biomarker.

In addition, the culture supernatants of BGC-823 and GES-1 cells were collected at 24, 48, and 72 h of culture (3000 cells per well) and the levels of potential biomarkers were determined as described above.

### RT-qPCR

RT-qPCR was utilized to detect the mRNA expression levels of potential biomarkers in the four types of GC cells and the control GES-1 cells. Cells were harvested at their logarithmic growth phase, and total RNA was extracted using a commercial kit provided by Yeasen (China). RNA quantification was performed using Nanodrop 2000 (Thermo Fisher Scientific, USA). Reverse transcription and quantitative PCR amplification of the target genes were performed using Yeasen (China) kits. PCR amplification primers were designed and synthesized by Sangon Biotech Co., Ltd. (Shanghai, China).

### Plasmid extraction

The DH-5α glycerol bacteria harboring the overexpressed plasmid were provided by Shanghai Genechem Co., Ltd (China). After carefully homogenizing the bacterial suspension, we diluted it by 100, 1000, and 10,000, respectively, and spread the dilutions onto an LB solid culture plates containing ampicillin. The plates were incubated at 37℃ for 12–16 h. Following incubation, individual colonies were carefully selected and inoculated into LB liquid medium containing ampicillin. These cultures were incubated with shaking at 37℃ and 220 rpm for 12–16 h to amplify bacterial growth. Finally, plasmid extraction was performed using a kit from TIANGEN Biotech(Beijing)Co., Ltd (China).

### Cell culture and transfection

Human gastric cancer cell lines (AGS, MKN45, and HGC27) and normal gastric mucosal epithelial cell line (GES1) were purchased from the Cell Bank of the Chinese Academy of Sciences. The human gastric cancer cell line BGC823 was purchased from Shanghai FuHeng Biology Co., Ltd. (China). These cells were routinely cultured at 37 °C with 5% CO_2_. The cell lines GES-1, HGC27, MKN45, and BGC823 were cultured in RPMI-1640 medium with 10% fetal bovine serum (GIBCO USA), while AGS cells were cultured in DMEM-F12 medium with 10% fetal bovine serum.

To regulate the mRNA expression of potential biomarkers in GC cells, we used small interfering RNA (siRNA) for downregulation and overexpression plasmid for upregulation. GC cells were seeded in cell plates and cultured to 40% confluence. The siRNA sequences and over-expressed plasmids targeting the potential biomarkers were transfected using LIPO3000 reagent (Invitrogen, USA). Following transfection, the cells were incubated for 24–72 h for mRNA level analysis of the potential biomarkers. The siRNA sequences were designed and synthesized by a company (Genepharma, China).

### Cell counting Kit-8

After a 24-hour transfection period, the cells underwent trypsin digestion and were resuspended in a complete culture medium to achieve a concentration of 2 × 10^4 cells/ml. Subsequently, 100 μl aliquots of this cell suspension were dispensed into a 96-well plate and incubated under standard cell culture conditions. At specific time intervals (0, 24, 48, 72, and 96 h), 10 μl of Cell Counting Kit-8 (CCK-8) reagent (GLPBIO, USA) was added to each well, followed by 2 h of incubation in the dark. Finally, the optical density (OD) was measured at 450 nm using a microplate reader (Thermo Fisher Scientific, USA) and utilized to construct cell proliferation curves.

### Cell clone formation

After a 24-hours transfection period, the cells were detached using trypsin and diluted to achieve a density of 700 cells/ml. Subsequently, 2 ml aliquots of the cell suspension were dispensed into each well of a 6-well plate, and the cultures were maintained with regular medium changes every three days. The incubation period lasted for 14 days or until the majority of individual cell clones exceeded 50 cells. Once the culturing was terminated, the cells were washed with PBS, fixed with 4% paraformaldehyde for 30 min, stained with crystal violet for 10–20 min, and finally rinsed thoroughly with PBS and dried naturally.

### Statistical analysis

The statistical analysis was carried out using R software (version 4.2.2) and GraphPad Prism 8.0 software. Continuous variables are presented as Mean ± standard deviation. For comparisons between groups, t-tests were utilized when the data followed a normal distribution. Wilcoxon rank-sum tests were employed for data that did not adhere to a normal distribution. *P* < 0.05 was statistical significance.

## Results

### Candidate biomarkers identified by trans-omics analysis

In our previous proteomic dataset utilizing aptamers, we identified 236 differentially expressed serum proteins specific to GC (Supplementary Table 1). Their corresponding genes were found in the UniProt database. Analysis of the TCGA-STAD transcriptomic dataset uncovered 10,637 differentially expressed genes (Supplementary Fig. 1). There were 119 overlapping DEGs between the two datasets (Supplementary Table 2, Supplementary Fig. 2).

**Fig. 1:**
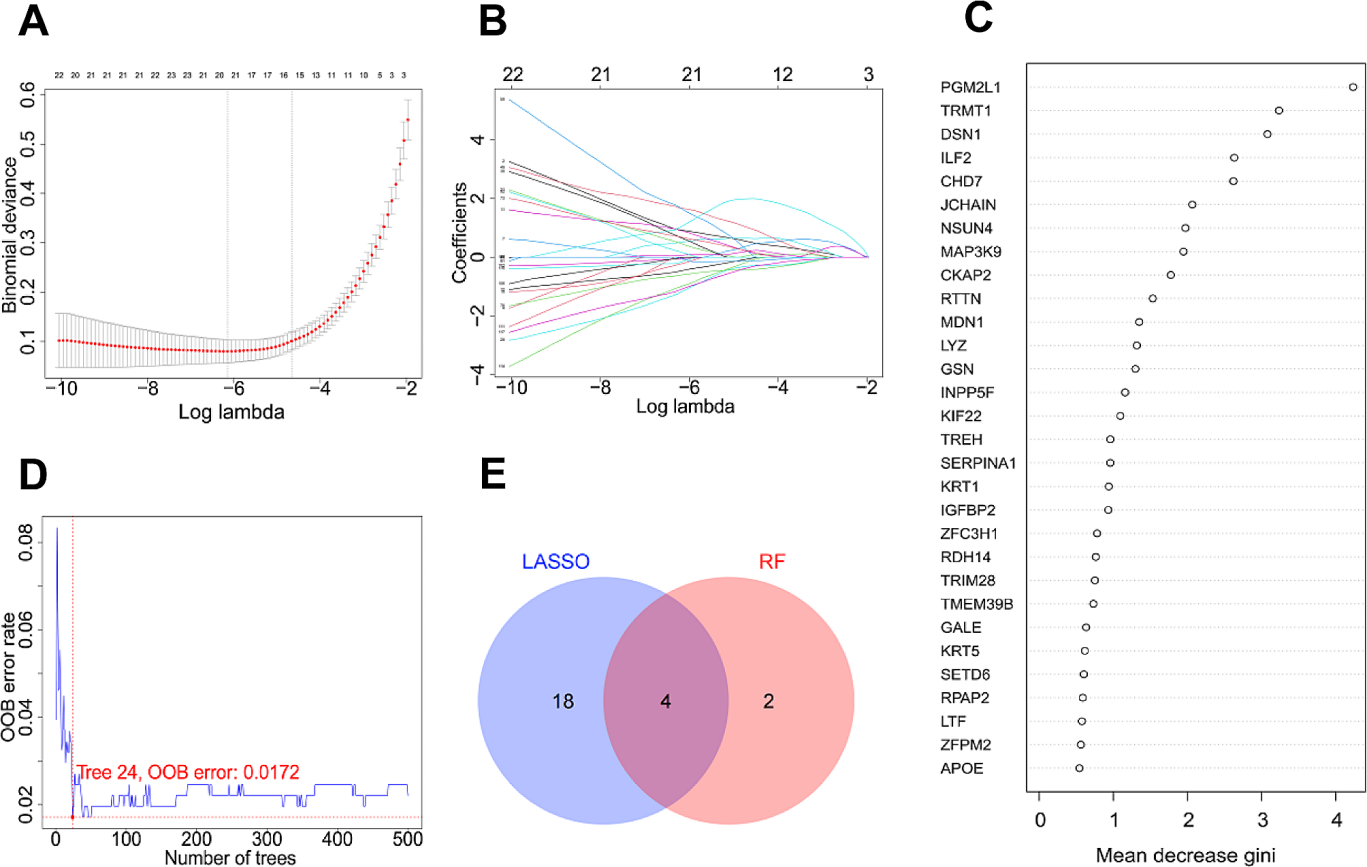
Identification of candidate biomarkers for gastric cancer. **A, B**: The selection of key overlapping differentially expressed genes (DEGs) by least absolute shrinkage and selection operator (LASSO) regression analysis. **C, D**: The selection of key overlapping DEGs by random forest (RF) analysis. **E**: Venn diagram of key overlapping DEGs selected by LASSO regression and RF analysis

To identify key DEGs, we employed LASSO regression and random forest analyses on the overlapping DEG set. The LASSO regression with λ = 0.002 selected 22 key DEGs (Fig. 1A, B). The random forest analysis pinpointed the 24th decision tree with the lowest OOB error rate of 0.0172 for GC vs. normal sample classification, in which six genes had importance scores > 2, as evaluated by Gini importance (Fig. 1C, D). The LASSO regression and random forest selection identified four DEGs that overlapped, namely interleukin enhancer-binding factor 2 (ILF2), immunoglobulin J chain (JCHAIN), chromodomain helicase DNA-binding protein 7 (CHD7), and phosphoglucomutase-2-like 1 (PGM2L1) (Fig. 1E), which were designated as candidate biomarkers for GC.

### Validation of candidate biomarker expression

**Fig. 2:**
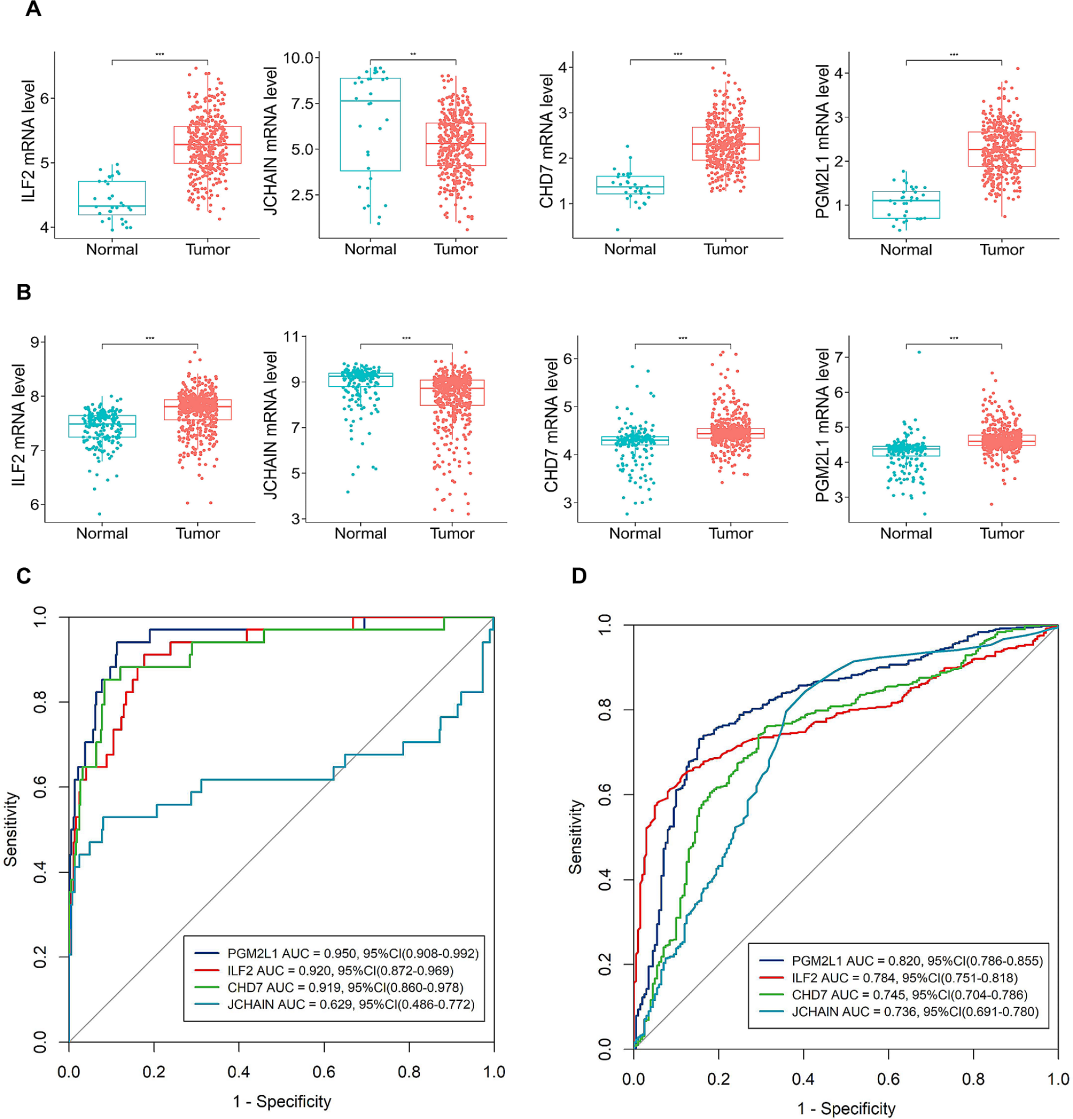
Expression and diagnosis value of the four candidate biomarkers in gastric cancer and normal control tissues. * *P* < 0.05, ** *P* < 0.01, *** *P* < 0.001. **A**: STAD dataset from The Cancer Genome Atlas database; **B**: Merged datasets of GSE27342, GSE54129 and GSE66229 datasets from the Gene Expression Omnibus (GEO) database. **C**: STAD dataset from The Cancer Genome Atlas database; **D**: Merged dataset of GSE27342, GSE54129 and GSE66229 datasets from the Gene Expression Omnibus (GEO) database; AUC: area under the curve

In the TCGA-STAD dataset used for internal validation, ILF2 (Interleukin enhancer-binding factor 2), CHD7 (Chromodomain-helicase-DNA-binding protein 7), and PGM2L1 (Glucose 1,6-bisphosphate synthase) demonstrated significantly higher mRNA expression levels in GC than in control tissues, whereas JCHAIN (Immunoglobulin J chain) showed significantly lower mRNA expression levels (Fig. 2A). In the external validation using the merged GSE dataset, these candidate biomarkers showed the same trends in mRNA expression levels and significant differences between GC tissues and adjacent normal tissues (Fig. 2B). Before analysis, the external validation datasets (GSE27342, GSE54129, and GSE66229) retrieved from the GEO database underwent standardization, resulting in consistent gene expression distributions (Supplementary Fig. 3A). Following batch-effect correction, the differences among these datasets in the principal component space were significantly reduced (Supplementary Fig. 3B, C), indicating successful merging of the three GES datasets.

### Diagnostic value of candidate biomarkers for GC

We assessed the diagnostic significance of mRNA levels for the four potential biomarkers in GC using the receiver operating characteristic curve (ROC). In the TCGA-STAD dataset, the AUROCs for diagnosing GC with PGM2L1, ILF2, CHD7, and JCHAIN were 0.950, 0.920, 0.919, and 0.629, respectively (Fig. 2C). In the external validation dataset (the merged GSE dataset), the AUROCs for PGM2L1, ILF2, CHD7, and JCHAIN in diagnosing GC were 0.820, 0.784, 0.745, and 0.736, respectively (Fig. 2D). These results showed that PGM2L1, ILF2, and CHD7 (but not JCHAIN) all had AUROCs greater than 0.7 in both the datasets, highlighting their diagnostic potential for GC and were therefore selected as valuable biomarkers for subsequent studies.

### Associations of the valuable candidate biomarkers with tumor immunity

**Fig. 3:**
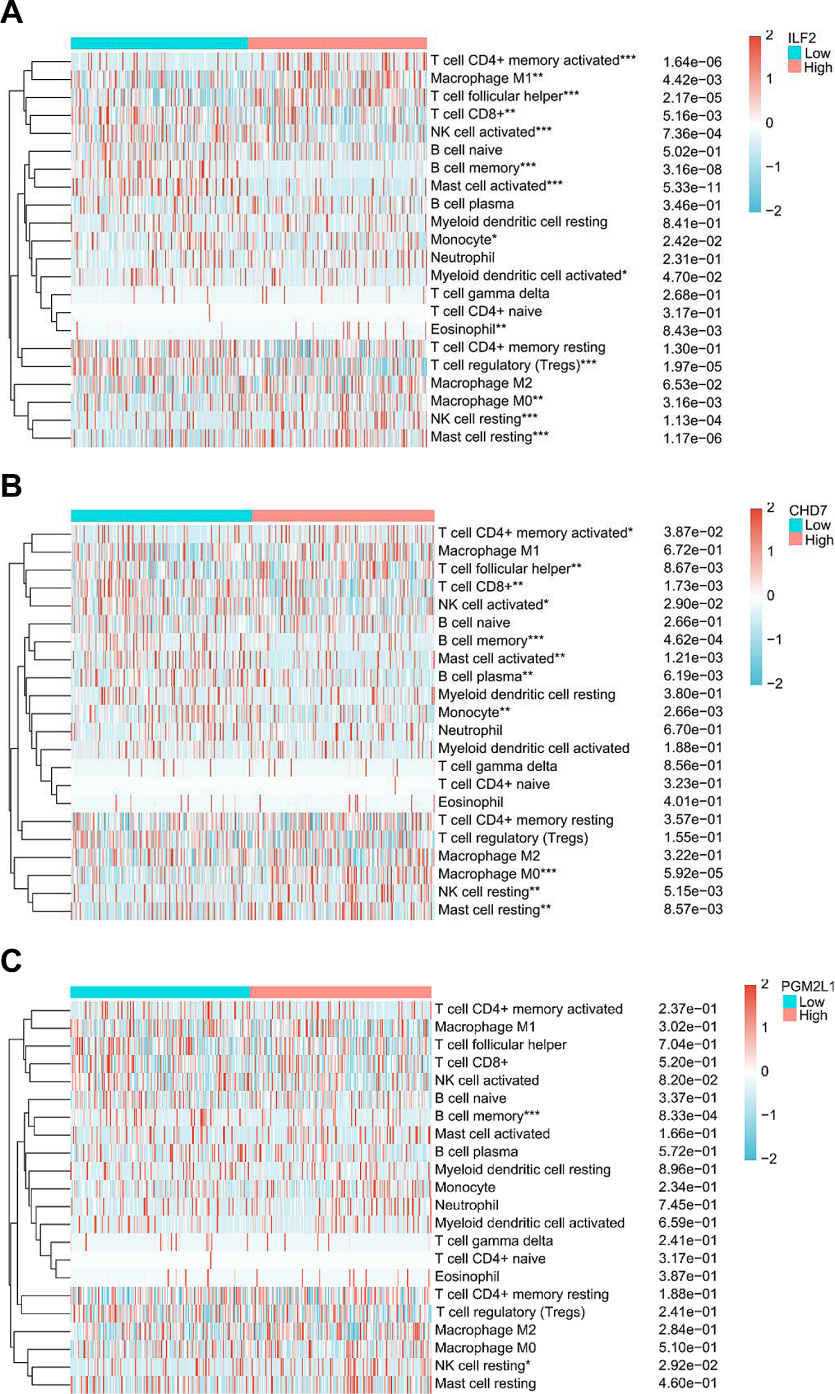
Heatmaps of immune cell scores and comparisons between high and low biomarker expression groups. *P < 0.05, **P < 0.01, ***P < 0.001. **A**: ILF2; **B**: CHD7; **C**: PGM2L1

**Fig. 4:**
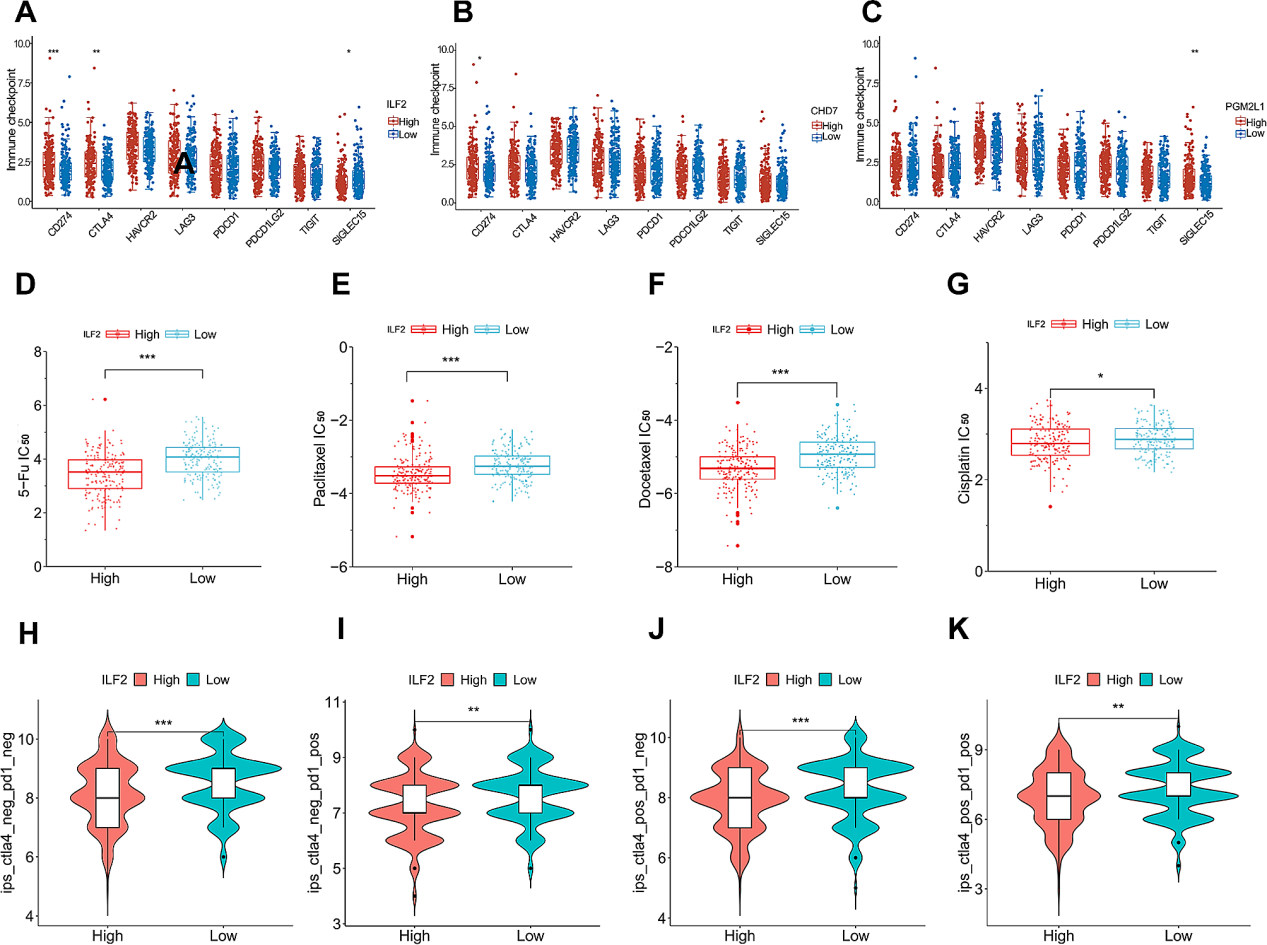
Bioinformatics analyses of ILF2 in gastric cancer. *P < 0.05, **P < 0.01, ***P < 0.001. **A-C**: Comparison of immune checkpoint-related gene expression levels between the high and low expression groups of biomarkers. **D-G**: Comparison of sensitivity to anticancer drugs between the high and low ILF2 expression groups. **H-K**: Comparison of response to immunotherapy between the high and low ILF2 expression groups; IPS, the Immunophenoscore; POS, positive; NEG, negative

Using CIBERSORT analysis, we compared the immune cell infiltration status in GC tissues between high and low expression levels of each biomarker. The results showed that ILF2, CHD7, and PGM2L1 were significantly associated with 14, 11, and 2 of 22 types of infiltrating immune cells, respectively (Fig. 3A-C). From the transcriptome data in the TCGA-STAD dataset, we extracted expression levels of 8 immune checkpoint genes (TIGIT, HAVCR2, CD274, SIGLEC15, LAG3, PDCD1, PDCD1LG2, and CTLA4). Comparing differences in their expression between high and low levels of each biomarker, we found that ILF2 expression was significantly associated with CD274, CTLA4, and SIGLEC15; CHD7 was significantly associated with CD274; and PGM2L1 was significantly associated with SIGLEC15 (Fig. 4A-C). These findings suggest that, among the three biomarkers, ILF2 exhibits the strongest association with tumor immunity in GC, involving multiple immune checkpoint genes. Therefore, ILF2 was selected as a potential biomarker for subsequent studies.

### Associations of ILF2 with immunotherapy and chemotherapy responses

We utilized the GDSC database to analyze the variations in response to standard chemotherapy drugs (5-fluorouracil, paclitaxel, docetaxel, and cisplatin) [26] among gastric cancer patients with different levels of ILF2 expression. The findings indicate that the IC_50_s were lower in the high-level ILF2 group compared with the low-level ILF2 group. Hence, GC with increased ILF2 levels exhibits enhanced response to these drugs (Fig. 4D-G). Additionally, using the TCIA database, we calculated CTLA4- and PD-1-based immunophenotype scores (IPS) for GC patients and compared the response to anti-CTLA4 and anti-PD-1 immunotherapy between patients with high and low ILF2 expression levels. The results indicated that individuals with elevated ILF2 levels exhibited significantly attenuated responses to CTLA4 and PD-1 inhibitors (Fig. 4H-K).

### Clinical validation of the diagnostic significance of serum ILF2 levels in gastric cancer

**Fig. 5:**
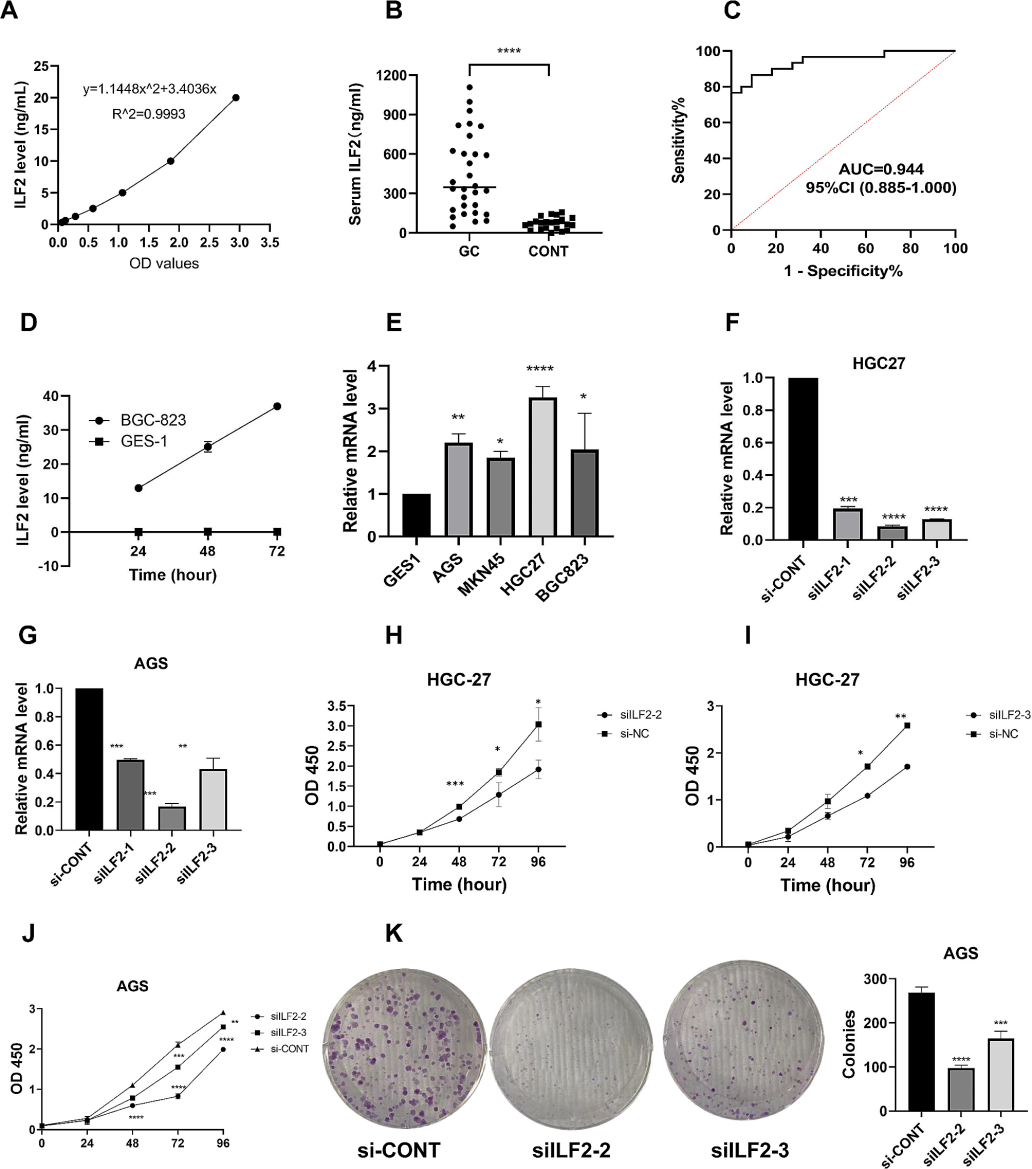
Clinical and functional validation of ILF2. **A**: The standard curve for the detection of ILF2 by enzyme-linked immunosorbent assay. **B**: Serum ILF2 levels and comparison between gastric cancer and control group. **C**: The receiver operating characteristic curve of serum ILF2 levels for the diagnosis of gastric cancer. **D**: ILF2 levels in cell culture supernatants. **E**: Relative mRNA expression levels of ILF2 in different gastric cancer cell lines; **F, G**: The effect of siRNA-mediated ILF2 knockdown on mRNA expression in HGC-27 and AGS cells; **H-J**: Comparisons of HGC-27 cell proliferation between siRNA-mediated ILF2 knockdown and control groups. **K**: The effect of siRNA-mediated ILF2 knockdown on clone formation in AGS cells. AUC, Area Under Curve; CI, Confidence Interval; OD, Optical Density

Utilizing ELISA (its standard curve shown in Figure 5A), serum levels of ILF2 protein were measured and notably elevated in the GC group when compared to the control group (443.23 ± 303.29 ng/mL vs. 72.31 ± 45.18 ng/mL, *P* < 0.0001) (Fig. 5, B). ILF2 levels were valuable for diagnosing GC with an AUROC of 0.944 (Fig. 5C). The baseline information of the patients is shown in Supplementary Table S3.

### Detection of ILF2 expression in the supernatant of gastric cancer cell culture

The presence of ILF2 protein was observed in the culture supernatants of BGC823 GC cells, and its concentration increased as the culture time progressed. However, it was not detected in the supernatants of GES-1 gastric epithelial cells (Fig. 5D), indicating that gastric cancer cells can secret ILF2.

### Expression levels of ILF2 in GC cells

The ILF2 mRNA expression was detected by RT-qPCR in four GC cell lines (AGS, MKN45, HGC27, and BGC823) and one control cell line (GES-1). The primer sequences used for the PCR amplification of ILF2 were 5’- GGGGAACAAAGTCGTGGAAAG-3’ (forward) and 5’- CCAGTTTCGTTGGTCAGCA-3’ (reverse). The results indicated a significant upregulation of ILF2 mRNA expression in all four GC cell lines compared to the control (Fig. 5E).

### Effect of ILF2 knockdown on GC cell growth

We knocked down ILF2 transcription using siRNAs in HGC27 and AGS cells. Three ILF2 mRNA-specific siRNA sequences (siILF2-1, siILF2-2, and siILF2-3) and one control sequence (siILF2-CONT) were employed in the knockdown experiments. Their nucleic acid sequences (sense) were as follows:

siILF2-1: 5’-GAUAGUAACACCUUCAGAATT-3’.

siILF2-2: 5’-CUUUGUACCACAUAUCCCATT-3’.

siILF2-3: 5’-GCUACAGUGAAGAUUCUCATT-3’.

siILF2-CONT: 5’-UUUCUCCGAACGUGUCACGUTT-3’.

The findings indicated that the siRNA depletion notably reduced the ILF2 mRNA levels (Fig. 5F, G**).** Among the three siRNAs, siILF2-2 exhibited the most effective knockdown. In CCK-8 experiments, there was a notable reduction in cell proliferation at 48, 72, and 96 h in HGC27 cells and 72 and 96 h in AGS cells transfected with siILF2-2 and siILF2-3 compared to the control (Fig. 5H-J). In clone formation experiments (Fig. 5K), the number of AGS cell clones with knocked-down ILF2 decreased significantly.

### Effect of ILF2 overexpression on the growth of gastric cancer cells

**Fig. 6:**
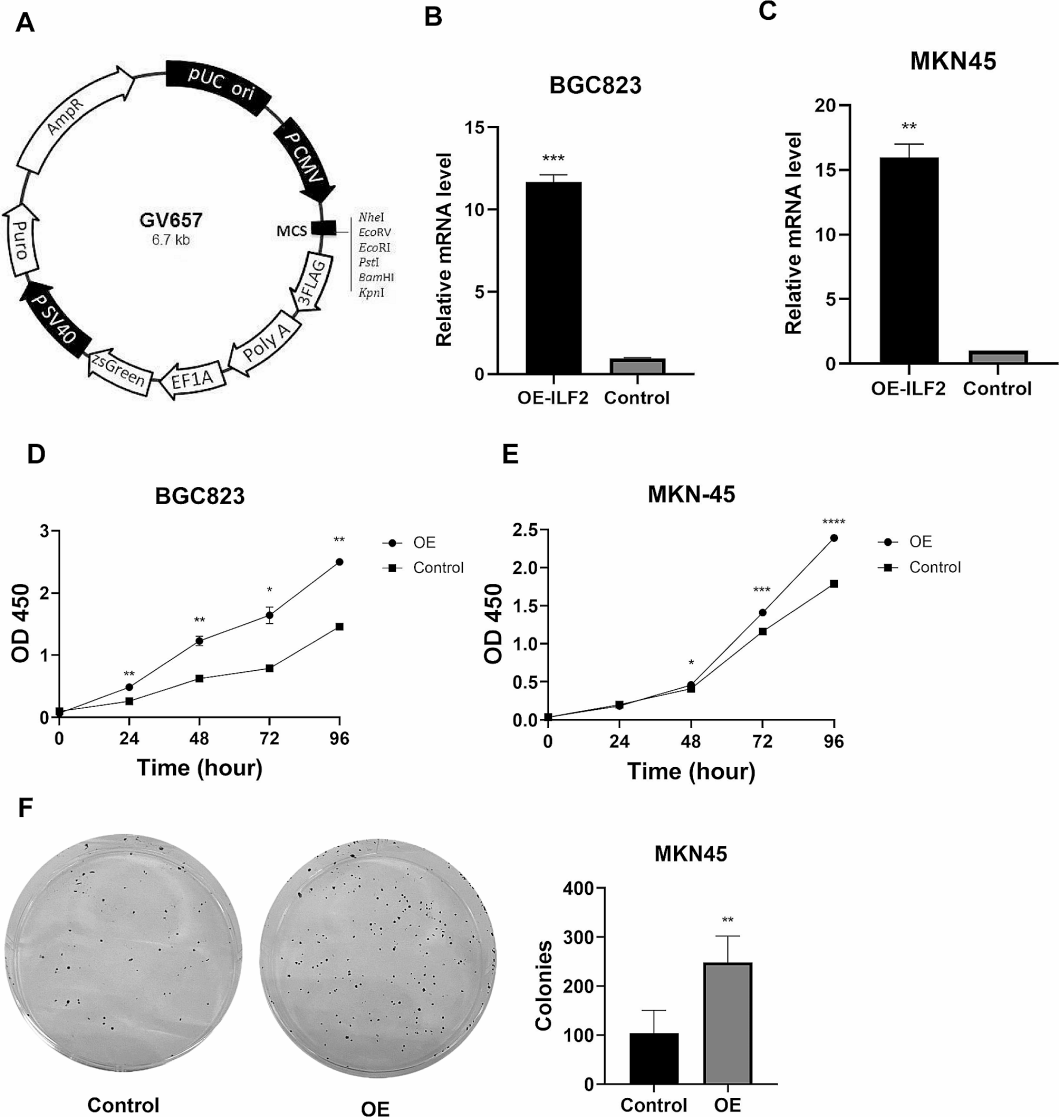
The effect of overexpressing ILF2 on the function of gastric cancer cells. **A** The vector of ILF2. **B, C** The effect of ILF2 overexpression on mRNA expression in BGC823 and MKN45 cells. **D, E**: Comparisons of cell proliferation between ILF2 over-expressed groups and control groups in BGC823 and MKN45 cells. **F.** The effect of ILF2 over-expressed on clone formation in MKN45 cells. OE, over expression

The ILF2 overexpression plasmid was designed by Shanghai Genechem Co., Ltd (China), and vector details are presented in Fig. 6A. The results indicated that ILF2 overexpression significantly increased the level of ILF2 mRNA, as demonstrated in Fig. 6B, C. In the CCK-8 assay, BGC823 and MKN45 cells with overexpression of ILF2 exhibited significantly enhanced proliferative capacity compared to the control group (Fig. 6D-E). Additionally, In the clone formation experiment (Fig. 6F), the number of MKN45 cell clones overexpressing ILF2 increased significantly.

## Discussion

Through the cross-analysis of our aptamer-based serum proteomic dataset and the TCGA-STAD tissue transcriptomic dataset, we have discovered four candidate biomarkers for GC: ILF2, JCHAIN, PGM2L1, and CHD7. Except for JCHAIN, all the candidate biomarkers proved useful in diagnosing GC in both the TCGA-STAD and the merged GSE dataset. Among the three candidate biomarkers with significant value, ILF2 stood out as it exhibited a strong correlation with the infiltration of immune cells and the expression of immune checkpoint genes in GC. As a result, ILF2 was selected as a promising diagnostic biomarker for GC. Subsequent studies showed that ILF2 was significantly associated with GC in terms of chemotherapy sensitivity and immunotherapy response. In the clinical validation study, ILF2 protein levels were significantly elevated in GC serum samples and exhibited good diagnostic performance for GC. In vitro experiments demonstrated that GC cells exhibited elevated levels of ILF2 expression and the ability to secrete ILF2. The suppression of ILF2 expression impeded the proliferation and clone formation of GC cells, while overexpression of ILF2 promoted these processes.

ILF2, also known as nuclear factor 45 (NF45), interacts with different partners to play a role in controlling gene expression at both the transcriptional and post-transcriptional stages. By binding to the interleukin-2 enhancer, ILF2 promotes the transcription of interleukin-2 in activated T cells [28]. ILF2 can interact with lncRNA, a type of ribonucleic acid that does not code for proteins, to control the maturation of miRNA [29]. Additionally, ILF2 can also interact with nuclear proteins like YB-1 to regulate the processes of RNA splicing and degradation [30].

ILF2 gene amplification and overexpression in different human cancers promote cellular processes such as cell growth, programmed cell death, and invasion [31]. ILF2 is upregulated in several malignancies, including liver and lung cancer, where its high expression stimulates malignant phenotypes in liver cells [32, 33] and interacts with E2F transcription factor 1 (E2F1) to promote lung cancer progression [34]. The suppression of ILF2 expression leads to improved prognosis in breast cancer [35].

Reports on the study of ILF2 in GC are limited. Yin et al. [36] first detected the ILF2 expression levels in both tumor and paired normal tissues of GC using qRT-PCR, Western blot, and immunohistochemistry, and found that ILF2 was predominantly localized to the nuclei of gastric cancer cells, and also exhibited a low level of expression in the cytoplasm. Furthermore, the overexpression of ILF2 in tumor tissues had substantial prognostic significance in gastric cancer. Arai et al. [37] further identified ILF2 as a notable protein present in both gastric cancer tissues and cell lines, functioning as a DNA damage response (DDR) protein. In addition, the ILF2/ILF3 complex dynamically regulates the expression of lncRNA ELF3-AS1 and transcription factor ELF3, thereby influencing GC metastasis [38]. However, the impact of ILF2 on GC diagnosis and therapy and the association of ILF2 with GC cell growth and tumor immunity remain unclear.

In the present study, we found that ILF2 had a good diagnostic value for GC. The high expression of ILF2 at the transcriptional level in GC tissues effectively distinguished cancer tissues from control tissues, which was confirmed not only in the discovery dataset (TCGA-STAD dataset) but also in the large-sample validation dataset merged from three independent GSE datasets of GC. More importantly, we also detected an elevated level of ILF2 in the serum samples of GC patients, approximately 6-fold higher than that of control samples. The AUROC for the diagnosis of GC reached 0.944. Given the absence of a valuable serum biomarker for GC and the ease of collecting serum samples in clinical practice, our discovery suggests a promising opportunity for clinical application and warrants additional investigation.

The findings of this research offer a solid biological foundation for the considerable diagnostic importance of ILF2 in GC. Through bioinformatics analysis, we discovered a strong correlation between ILF2 expression levels in GC tissues and tumor immunity, encompassing immune cell infiltration, expression of immune checkpoint genes, immunotherapy response, as well as chemosensitivity. In vitro experiments demonstrated that GC cell lines overexpressing ILF2 secreted the protein extracellularly and exhibited different chemosensitivity based on ILF2 expression levels. Moreover, the knockdown of ILF2 expression inhibited cell proliferation and colony formation, and overexpression of ILF2 promoted the proliferation and colony formation of gastric cancer cells. However, the question here is how ILF2, a nuclear protein, is secreted outside the cell. Future studies are required to clarify the unclear underlying mechanism.

In this study, we used multiple datasets, including the TCGA-STAD dataset, the GEO dataset, and our serum proteomics dataset and serum specimen dataset. The good representation of datasets from different sources used in the same study favors obtaining research results with better generalization. However, some limitations of these datasets may cause bias; for example, the TCGA-STAD dataset has an imbalance in case numbers between gastric cancer and control groups, there are differences in RNA-Seq data between the GEO datasets (although compensated by data processing techniques), and our own serum sample dataset has a small sample size and insufficient control groups. Although we have successfully screened and validated ILF2 as a valuable biomarker for gastric cancer through rigorous multistep screening and validation, a larger sample size and multicenter validation of the results are still needed.

Moreover, further elucidation is needed in several areas, including clarifying the mechanism by which gastric cancer cells secrete ILF2 and investigating the impact of inhibiting the functional activity of extracellular ILF2 with neutralizing antibodies or small molecules (if available) on the biological behavior of gastric cancer, which will be crucial for revealing the therapeutic potential of ILF2. Additionally, in-depth research is required to understand how ILF2 regulates the biological behaviors of gastric cancer and assess the influence of ILF2 expression on migration, invasion phenotypes, and immune modulation in gastric cancer. To gain a deeper understanding of the role and exact regulatory mechanisms of ILF2 in gastric cancer, further in vivo and in vitro studies are essential.

## Conclusions

In this study, we utilized cross-omics analysis and identified ILF2 as a promising serum diagnostic marker for gastric cancer, exhibiting good diagnostic value and applicability. Bioinformatics analysis revealed a significant correlation between ILF2 and immune status and therapeutic response in gastric cancer. In vitro experiments confirmed that ILF2 is highly expressed in gastric cancer cells and influences cell growth. ILF2 levels were significantly elevated in the serum of gastric cancer patients, demonstrating its diagnostic significance. However, there are still several related issues that require clarification, including the secretion mechanism of ILF2 from gastric cancer cells and the precise mechanism by which ILF regulates the biological behaviors of gastric cancer. Furthermore, the diagnostic significance of serum ILF2 concentration for gastric cancer should be validated through large-sample and multicenter studies.

### Electronic supplementary material

Below is the link to the electronic supplementary material.

Supplementary Material 1

## Data Availability

The datasets used and/or analysed during the current study are available from the corresponding author on reasonable request.

## Abbreviations

5-FU: 5-Fluorouracil
AUROC: Receiver operating characteristic curve
CA125: Carbohydrate antigen 125
CA19-9: Carbohydrate antigen 19 − 9
CA72-4: Carbohydrate antigen 72 − 4
CCK-8: Cell Counting Kit-8
CEA: Carcinoembryonic antigen
CHD7: Chromodomain helicase DNA-binding protein 7
CIBERSORT: Cell-type Identification by Estimating Relative Subsets of RNA Transcripts
CTCs: Circulating tumor cells
ctDNA: Circulating tumor DNA
CTLA4: Cytotoxic T-lymphocyte-associated antigen 4
DEGs: Differentially expressed genes
ELISA: Enzyme-linked immunosorbent assay
G-17: Gastrin-17
GC: Gastric cancer
GDSC: The Cancer Drug Sensitivity Genomics
GEO: Gene Expression Omnibus
ILF2: Interleukin enhancer-binding factor 2
JCHAIN: Immunoglobulin J chain
OD: Optical density
OOB: Out-of-bag
PD-1: Programmed cell death protein-1
PGM2L1: Phosphoglucomutase-2-like 1
PGs: Pepsinogens
siRNA: Small interfering RNA
TCGA-STAD: The Cancer Genome Atlas-Stomach Adenocarcinoma
TCGA: The Cancer Genome Atlas
TCIA: The Cancer Immunome Atlas
TFF3: Trefoil factor 3
TMB: Tetra methyl benzidine
