# Supplementary material for: Integrating trans-omics, cellular experiments and clinical validation to identify ILF2 as a diagnostic serum biomarker and therapeutic target in gastric cancer
Source: BMC Cancer. 2024 Apr 15;24:465. doi: 10.1186/s12885-024-12175-z (PMC11017608; doi:10.1186/s12885-024-12175-z)
Supplement: Supplementary file 1 — Supplementary Material 1 [file 12885_2024_12175_MOESM1_ESM.docx]

**Supplementary Table 1** Differentially expressed proteins in our aptamer-based gastric cancer serum proteome and their corresponding genes in the Uniprot database

| **No** | **Protein names** | **Protein ID** | **Gene name** | **Gene ID** |
| --- | --- | --- | --- | --- |
|  | Alpha-1B-glycoprotein | P04217 | A1BG | ENSG00000121410 |
|  | Alpha-2-macroglobulin | P01023 | A2M | ENSG00000175899 |
|  | Actin, cytoplasmic 1 | P60709 | ACTB | ENSG00000075624 |
|  | Actin, gamma-enteric smooth muscle | P63267 | ACTG2 | ENSG00000163017 |
|  | Acylphosphatase-1 | P07311 | ACYP1 | ENSG00000119640 |
|  | Alpha-2-HS-glycoprotein | P02765 | AHSG | ENSG00000145192 |
|  | A-kinase anchor protein 6 | Q13023 | AKAP6 | ENSG00000151320 |
|  | Albumin | P02768 | ALB | ENSG00000163631 |
|  | Succinate-semialdehyde dehydrogenase -dependent succinic semialdehyde dehydrogenase) | P51649 | ALDH5A1 | ENSG00000112294 |
|  | Intestinal-type alkaline phosphatase | P09923 | ALPI | ENSG00000163295 |
|  | Protein AMBP | P02760 | AMBP | ENSG00000106927 |
|  | Ankyrin repeat and KH domain-containing protein 1 | Q8IWZ3 | ANKHD1 | ENSG00000131503 |
|  | Serum amyloid P-component | P02743 | APCS | ENSG00000132703 |
|  | Apolipoprotein A-I | P02647 | APOA1 | ENSG00000118137 |
|  | Apolipoprotein A-IV | P06727 | APOA4 | ENSG00000110244 |
|  | Apolipoprotein B-100 | P04114 | APOB | ENSG00000084674 |
|  | Apolipoprotein C-III | P02656 | APOC3 | ENSG00000110245 |
|  | Apolipoprotein E | P02649 | APOE | ENSG00000130203 |
|  | Apolipoprotein L1 | O14791 | APOL1 | ENSG00000100342 |
|  | Rho GTPase-activating protein 6 | O43182 | ARHGAP6 | ENSG00000047648 |
|  | Armadillo repeat-containing protein 8 | Q8IUR7 | ARMC8 | ENSG00000114098 |
|  | Ankyrin repeat and SOCS box protein 8 | Q9H765 | ASB8 | ENSG00000177981 |
|  | Argininosuccinate synthase | P00966 | ASS1 | ENSG00000130707 |
|  | ATP synthase subunit alpha | P25705 | ATP5F1A | ENSG00000152234 |
|  | Zinc-alpha-2-glycoprotein | P25311 | AZGP1 | ENSG00000160862 |
|  | Filensin | Q12934 | BFSP1 | ENSG00000125864 |
|  | Vesicle transport protein SEC20 | Q12981 | BNIP1 | ENSG00000113734 |
|  | Protein bassoon | Q9UPA5 | BSN | ENSG00000164061 |
|  | Blood vessel epicardial substance | Q8NE79 | BVES | ENSG00000112276 |
|  | Complement C1q subcomponent subunit C | P02747 | C1QC | ENSG00000159189 |
|  | Complement C1s subcomponent | P09871 | C1S | ENSG00000182326 |
|  | Complement C3 | P01024 | C3 | ENSG00000125730 |
|  | Complement C4-A | P0C0L4 | C4A | ENSG00000244731 |
|  | C4b-binding protein alpha chain | P04003 | C4BPA | ENSG00000123838 |
|  | Complement C5 | P01031 | C5 | ENSG00000106804 |
|  | Complement component C7 | P10643 | C7 | ENSG00000112936 |
|  | Complement component C8 beta chain | P07358 | C8B | ENSG00000021852 |
|  | Complement component C8 gamma chain | P07360 | C8G | ENSG00000176919 |
|  | Complement component C9 | P02748 | C9 | ENSG00000113600 |
|  | Caprin-2 | Q6IMN6 | CAPRIN2 | ENSG00000110888 |
|  | Coiled-coil domain-containing protein 25 | Q86WR0 | CCDC25 | ENSG00000147419 |
|  | PAT complex subunit CCDC47 | Q96A33 | CCDC47 | ENSG00000108588 |
|  | Monocyte differentiation antigen CD14 | P08571 | CD14 | ENSG00000170458 |
|  | CD5 antigen-like | O43866 | CD5L | ENSG00000073754 |
|  | Cilia- and flagella-associated protein 74 | Q9C0B2 | CFAP74 | ENSG00000142609 |
|  | Complement factor H-related protein 1 | Q03591 | CFHR1 | ENSG00000244414 |
|  | Complement factor I | P05156 | CFI | ENSG00000205403 |
|  | Chromodomain-helicase-DNA-binding protein 1 | O14646 | CHD1 | ENSG00000153922 |
|  | Chromodomain-helicase-DNA-binding protein 7 | Q9P2D1 | CHD7 | ENSG00000171316 |
|  | Carbohydrate sulfotransferase 12 | Q9NRB3 | CHST12 | ENSG00000136213 |
|  | Lipopolysaccharide-binding protein | P18428 | LBP | ENSG00000129988 |
|  | Apolipoprotein | P08519 | LPA | ENSG00000198670 |
|  | Lactotransferrin | P02788 | LTF | ENSG00000012223 |
|  | Lysozyme C | P61626 | LYZ | ENSG00000090382 |
|  | Microtubule-actin cross-linking factor 1, isoforms 1/2/3/4/5 | Q9UPN3 | MACF1 | ENSG00000127603 |
|  | Mitogen-activated protein kinase kinase kinase 9 | P80192 | MAP3K9 | ENSG00000006432 |
|  | Mannan-binding lectin serine protease 1 | P48740 | MASP1 | ENSG00000127241 |
|  | Mannan-binding lectin serine protease 2 | O00187 | MASP2 | ENSG00000009724 |
|  | DNA replication licensing factor MCM5 | P33992 | MCM5 | ENSG00000100297 |
|  | Midasin | Q9NU22 | MDN1 | ENSG00000112159 |
|  | MutS protein homolog 5 | O43196 | MSH5 | ENSG00000204410 |
|  | Myomesin-2 | P54296 | MYOM2 | ENSG00000036448 |
|  | Nocturnin | Q9UK39 | NOCT | ENSG00000151014 |
|  | 5-methylcytosine rRNA methyltransferase NSUN4 | Q96CB9 | NSUN4 | ENSG00000117481 |
|  | Centriole and centriolar satellite protein OFD1 | O75665 | OFD1 | ENSG00000046651 |
|  | Olfactory receptor 2B2 | Q9GZK3 | OR2B2 | ENSG00000168131 |
|  | Alpha-1-acid glycoprotein 1 | P02763 | ORM1 | ENSG00000229314 |
|  | Alpha-1-acid glycoprotein 2 | P19652 | ORM2 | ENSG00000228278 |
|  | Bifunctional 3'-phosphoadenosine 5'-phosphosulfate synthase 2 | O95340 | PAPSS2 | ENSG00000198682 |
|  | Propionyl-CoA carboxylase alpha chain | P05165 | PCCA | ENSG00000175198 |
|  | Glucose 1,6-bisphosphate synthase | Q6PCE3 | PGM2L1 | ENSG00000165434 |
|  | Peptidyl-prolyl cis-trans isomerase NIMA-interacting 4 | Q9Y237 | PIN4 | ENSG00000102309 |
|  | Prolactin-inducible protein | P12273 | PIP | ENSG00000159763 |
|  | Phospholipid transfer protein | P55058 | PLTP | ENSG00000100979 |
|  | Paraneoplastic antigen Ma2 | Q9UL42 | PNMA2 | ENSG00000240694 |
|  | Peroxiredoxin-2 | P32119 | PRDX2 | ENSG00000167815 |
|  | Protein kinase C delta type | Q05655 | PRKCD | ENSG00000163932 |
|  | cGMP-dependent protein kinase 1 | Q13976 | PRKG1 | ENSG00000185532 |
|  | Vitamin K-dependent protein S | P07225 | PROS1 | ENSG00000184500 |
|  | Proline-rich protein 23C | Q6ZRP0 | PRR23C | ENSG00000233701 |
|  | Cytoskeleton-associated protein 2 | Q8WWK9 | CKAP2 | ENSG00000136108 |
|  | ATP-dependent Clp protease ATP-binding subunit clpX-like | O76031 | CLPX | ENSG00000166855 |
|  | Clusterin | P10909 | CLU | ENSG00000120885 |
|  | Ceruloplasmin | P00450 | CP | ENSG00000047457 |
|  | Carboxypeptidase B2 | Q96IY4 | CPB2 | ENSG00000080618 |
|  | Carboxypeptidase D | O75976 | CPD | ENSG00000108582 |
|  | Rootletin | Q5TZA2 | CROCC | ENSG00000058453 |
|  | Dermcidin | P81605 | DCD | ENSG00000161634 |
|  | Death effector domain-containing protein | O75618 | DEDD | ENSG00000158796 |
|  | Disco-interacting protein 2 homolog C | Q9Y2E4 | DIP2C | ENSG00000151240 |
|  | GTP-binding protein Di-Ras2 | Q96HU8 | DIRAS2 | ENSG00000165023 |
|  | Dihydrolipoyllysine-residue succinyltransferase component of 2-oxoglutarate dehydrogenase complex | P36957 | DLST | ENSG00000119689 |
|  | DNA methyltransferase 1-associated protein 1 | Q9NPF5 | DMAP1 | ENSG00000178028 |
|  | Dipeptidase 2 | Q9H4A9 | DPEP2 | ENSG00000167261 |
|  | Desmoglein-1 | Q02413 | DSG1 | ENSG00000134760 |
|  | Kinetochore-associated protein DSN1 homolog | Q9H410 | DSN1 | ENSG00000149636 |
|  | Dual specificity protein phosphatase 6 | Q16828 | DUSP6 | ENSG00000139318 |
|  | Cytoplasmic dynein 2 heavy chain 1 | Q8NCM8 | DYNC2H1 | ENSG00000187240 |
|  | Enoyl-CoA hydratase | P30084 | ECHS1 | ENSG00000127884 |
|  | Extracellular matrix protein 1 | Q16610 | ECM1 | ENSG00000143369 |
|  | Eukaryotic translation initiation factor 4E-binding protein 2 | Q13542 | EIF4EBP2 | ENSG00000148730 |
|  | Engulfment and cell motility protein 2 | Q96JJ3 | ELMO2 | ENSG00000062598 |
|  | Electron transfer flavoprotein subunit alpha | P13804 | ETFA | ENSG00000140374 |
|  | Prothrombin | P00734 | F2 | ENSG00000180210 |
|  | Fibulin-5 | Q9UBX5 | FBLN5 | ENSG00000140092 |
|  | Ficolin-2 | Q15485 | FCN2 | ENSG00000160339 |
|  | Ficolin-3 | O75636 | FCN3 | ENSG00000142748 |
|  | Fermitin family homolog 3 | Q86UX7 | FERMT3 | ENSG00000149781 |
|  | Filaggrin-2 | Q5D862 | FLG2 | ENSG00000143520 |
|  | Filamin-A | P21333 | FLNA | ENSG00000196924 |
|  | Fibronectin | P02751 | FN1 | ENSG00000115414 |
|  | GRB2-associated-binding protein 2 | Q9UQC2 | GAB2 | ENSG00000033327 |
|  | UDP-glucose 4-epimerase | Q14376 | GALE | ENSG00000117308 |
|  | Glyceraldehyde-3-phosphate dehydrogenase, testis-specific | O14556 | GAPDHS | ENSG00000105679 |
|  | Ribosome-releasing factor 2 | Q969S9 | GFM2 | ENSG00000164347 |
|  | ADP-ribosylation factor-binding protein GGA1 | Q9UJY5 | GGA1 | ENSG00000100083 |
|  | Glycine dehydrogenase | P23378 | GLDC | ENSG00000178445 |
|  | Glucose-6-phosphate isomerase | P06744 | GPI | ENSG00000105220 |
|  | Uracil nucleotide/cysteinyl leukotriene receptor | Q13304 | GPR17 | ENSG00000144230 |
|  | Gelsolin | P06396 | GSN | ENSG00000148180 |
|  | Histone H1.3 | P16402 | H1-3 | ENSG00000124575 |
|  | Histone H2A type 1-B/E | P04908 | H2AC4 | ENSG00000278463 |
|  | Hemoglobin subunit beta | P68871 | HBB | ENSG00000244734 |
|  | Hemoglobin subunit gamma-1 | P69891 | HBG1 | ENSG00000213934 |
|  | Haptoglobin | P00738 | HP | ENSG00000257017 |
|  | Heat shock 70 kDa protein 1-like | P34931 | HSPA1L | ENSG00000204390 |
|  | E3 ubiquitin-protein ligase HUWE1 | Q7Z6Z7 | HUWE1 | ENSG00000086758 |
|  | Insulin-like growth factor-binding protein complex acid labile subunit | P35858 | IGFALS | ENSG00000099769 |
|  | Insulin-like growth factor-binding protein 2 | P18065 | IGFBP2 | ENSG00000115457 |
|  | Immunoglobulin heavy constant alpha 1 | P01876 | IGHA1 | ENSG00000211895 |
|  | Serine protease 1 | P07477 | PRSS1 | ENSG00000204983 |
|  | Ras-related protein Rab-25 | P57735 | RAB25 | ENSG00000132698 |
|  | Retinol dehydrogenase 14 | Q9HBH5 | RDH14 | ENSG00000240857 |
|  | RAB11-binding protein RELCH | Q9P260 | RELCH | ENSG00000134444 |
|  | E3 ubiquitin-protein ligase RNF123 | Q5XPI4 | RNF123 | ENSG00000164068 |
|  | Rho-associated protein kinase 2 | O75116 | ROCK2 | ENSG00000134318 |
|  | Putative RNA polymerase II subunit B1 CTD phosphatase RPAP2 | Q8IXW5 | RPAP2 | ENSG00000122484 |
|  | Rotatin | Q86VV8 | RTTN | ENSG00000176225 |
|  | Protein S100-A9 | P06702 | S100A9 | ENSG00000163220 |
|  | Serum amyloid A-1 protein | P0DJI8 | SAA1 | ENSG00000173432 |
|  | Serum amyloid A-2 protein | P0DJI9 | SAA2 | ENSG00000134339 |
|  | Serine--tRNA ligase synthetase) | Q9NP81 | SARS2 | ENSG00000104835 |
|  | Sterol carrier protein 2 | P22307 | SCP2 | ENSG00000116171 |
|  | P-selectin glycoprotein ligand 1 | Q14242 | SELPLG | ENSG00000110876 |
|  | Septin-12 | Q8IYM1 | SEPTIN12 | ENSG00000140623 |
|  | Alpha-1-antitrypsin | P01009 | SERPINA1 | ENSG00000197249 |
|  | Alpha-1-antichymotrypsin | P01011 | SERPINA3 | ENSG00000196136 |
|  | Thyroxine-binding globulin | P05543 | SERPINA7 | ENSG00000123561 |
|  | Antithrombin-III | P01008 | SERPINC1 | ENSG00000117601 |
|  | Heparin cofactor 2 | P05546 | SERPIND1 | ENSG00000099937 |
|  | Plasma protease C1 inhibitor | P05155 | SERPING1 | ENSG00000149131 |
|  | N-lysine methyltransferase SETD6 | Q8TBK2 | SETD6 | ENSG00000103037 |
|  | Protein Shroom3 | Q8TF72 | SHROOM3 | ENSG00000138771 |
|  | Spectrin alpha chain, non-erythrocytic 1 | Q13813 | SPTAN1 | ENSG00000197694 |
|  | Storkhead-box protein 1 | Q6ZVD7 | STOX1 | ENSG00000165730 |
|  | Succinate--CoA ligase subunit alpha | P53597 | SUCLG1 | ENSG00000163541 |
|  | Nesprin-1 | Q8NF91 | SYNE1 | ENSG00000131018 |
|  | Transcription factor 21 | O43680 | TCF21 | ENSG00000118526 |
|  | Transcobalamin-2 | P20062 | TCN2 | ENSG00000185339 |
|  | Beta-tectorin | Q96PL2 | TECTB | ENSG00000119913 |
|  | Immunoglobulin heavy constant gamma 1 | P01857 | IGHG1 | ENSG00000211896 |
|  | Immunoglobulin heavy constant gamma 2 | P01859 | IGHG2 | ENSG00000211893 |
|  | Immunoglobulin heavy constant mu | P04220 | IGHM | ENSG00000211899 |
|  | Immunoglobulin heavy variable 1-2 | P23083 | IGHV1-2 | ENSG00000211934 |
|  | Immunoglobulin heavy variable 1-46 | P01743 | IGHV1-46 | ENSG00000211962 |
|  | Immunoglobulin heavy variable 3-23 | P01777 | IGHV3-23 | ENSG00000211949 |
|  | Immunoglobulin heavy variable 3-33 | P01772 | IGHV3-33 | ENSG00000211955 |
|  | Immunoglobulin kappa constant | P01834 | IGKC | ENSG00000211592 |
|  | Immunoglobulin kappa variable 1-17 | P01610 | IGKV1-17 | ENSG00000240382 |
|  | Immunoglobulin kappa variable 1-39 | P01597 | IGKV1-39 | ENSG00000242371 |
|  | Immunoglobulin kappa variable 1D-33 | P01605 | IGKV1D-33 | ENSG00000239975 |
|  | Immunoglobulin kappa variable 2D-40 | P01614 | IGKV2D-40 | ENSG00000251039 |
|  | Immunoglobulin kappa variable 3-11 | P04433 | IGKV3-11 | ENSG00000241351 |
|  | Immunoglobulin kappa variable 3-15 | P04207 | IGKV3-15 | ENSG00000244437 |
|  | Immunoglobulin kappa variable 3-20 | P06311 | IGKV3-20 | ENSG00000239951 |
|  | Immunoglobulin lambda constant 2 | P0CG06 | IGLC2 | ENSG00000211677 |
|  | Immunoglobulin lambda variable 1-40 | P01703 | IGLV1-40 | ENSG00000211653 |
|  | Immunoglobulin lambda variable 1-44 | P01699 | IGLV1-44 | ENSG00000211651 |
|  | Immunoglobulin lambda variable 3-21 | P80748 | IGLV3-21 | ENSG00000211662 |
|  | Immunoglobulin superfamily member 8 | Q969P0 | IGSF8 | ENSG00000162729 |
|  | Interleukin-17 receptor C | Q8NAC3 | IL17RC | ENSG00000163702 |
|  | Interleukin-1 receptor-like 1 | Q01638 | IL1RL1 | ENSG00000115602 |
|  | Interleukin enhancer-binding factor 2 | Q12905 | ILF2 | ENSG00000143621 |
|  | Phosphatidylinositide phosphatase SAC2 | Q9Y2H2 | INPP5F | ENSG00000198825 |
|  | Integrin alpha-V | P06756 | ITGAV | ENSG00000138448 |
|  | Inter-alpha-trypsin inhibitor heavy chain H1 | P19827 | ITIH1 | ENSG00000055957 |
|  | Inter-alpha-trypsin inhibitor heavy chain H2 | P19823 | ITIH2 | ENSG00000151655 |
|  | Inter-alpha-trypsin inhibitor heavy chain H3 | Q06033 | ITIH3 | ENSG00000162267 |
|  | Inter-alpha-trypsin inhibitor heavy chain H4 | Q14624 | ITIH4 | ENSG00000055955 |
|  | Intelectin-1 | Q8WWA0 | ITLN1 | ENSG00000179914 |
|  | Immunoglobulin J chain | P01591 | JCHAIN | ENSG00000132465 |
|  | Potassium voltage-gated channel subfamily H member 2 | Q12809 | KCNH2 | ENSG00000055118 |
|  | Kinesin-like protein KIF17 | Q9P2E2 | KIF17 | ENSG00000117245 |
|  | Kinesin-like protein KIF22 | Q14807 | KIF22 | ENSG00000079616 |
|  | Beta-klotho | Q86Z14 | KLB | ENSG00000134962 |
|  | Kininogen-1 | P01042 | KNG1 | ENSG00000113889 |
|  | Keratin, type II cytoskeletal 1 | P04264 | KRT1 | ENSG00000167768 |
|  | Keratin, type I cytoskeletal 10 | P13645 | KRT10 | ENSG00000186395 |
|  | Keratin, type I cytoskeletal 14 | P02533 | KRT14 | ENSG00000186847 |
|  | Keratin, type I cytoskeletal 16 | P08779 | KRT16 | ENSG00000186832 |
|  | Keratin, type II cytoskeletal 2 epidermal | P35908 | KRT2 | ENSG00000172867 |
|  | Keratin, type I cytoskeletal 27 | Q7Z3Y8 | KRT27 | ENSG00000171446 |
|  | Keratin, type I cuticular Ha1 | Q15323 | KRT31 | ENSG00000094796 |
|  | Keratin, type I cuticular Ha3-II | Q14525 | KRT33B | ENSG00000131738 |
|  | Keratin, type II cytoskeletal 4 | P19013 | KRT4 | ENSG00000170477 |
|  | Keratin, type II cytoskeletal 5 | P13647 | KRT5 | ENSG00000186081 |
|  | Keratin, type II cytoskeletal 6A | P02538 | KRT6A | ENSG00000205420 |
|  | Keratin, type II cytoskeletal 6B | P04259 | KRT6B | ENSG00000185479 |
|  | Keratin, type II cytoskeletal 6C | P48668 | KRT6C | ENSG00000170465 |
|  | Keratin, type I cytoskeletal 9 | P35527 | KRT9 | ENSG00000171403 |
|  | Serotransferrin | P02787 | TF | ENSG00000091513 |
|  | Thrombospondin-1 | P07996 | THBS1 | ENSG00000137801 |
|  | Toll-like receptor 9 | Q9NR96 | TLR9 | ENSG00000239732 |
|  | Transmembrane protein 39B | Q9GZU3 | TMEM39B | ENSG00000121775 |
|  | Trinucleotide repeat-containing gene 6B protein | Q9UPQ9 | TNRC6B | ENSG00000100354 |
|  | Trehalase | O43280 | TREH | ENSG00000118094 |
|  | E3 ubiquitin-protein ligase TRIM13 | O60858 | TRIM13 | ENSG00000204977 |
|  | Transcription intermediary factor 1-beta | Q13263 | TRIM28 | ENSG00000130726 |
|  | tRNA -N)-dimethyltransferase -N) methyltransferase) G26)dimethyltransferase) | Q9NXH9 | TRMT1 | ENSG00000104907 |
|  | Transient receptor potential cation channel subfamily M member 4 | Q8TD43 | TRPM4 | ENSG00000130529 |
|  | Transient receptor potential cation channel subfamily M member 5 | Q9NZQ8 | TRPM5 | ENSG00000070985 |
|  | Transthyretin | P02766 | TTR | ENSG00000118271 |
|  | Tuftelin | Q9NNX1 | TUFT1 | ENSG00000143367 |
|  | Polyubiquitin-C | P0CG48 | UBC | ENSG00000150991 |
|  | Intermembrane lipid transfer protein VPS13C | Q709C8 | VPS13C | ENSG00000129003 |
|  | Vitronectin | P04004 | VTN | ENSG00000109072 |
|  | von Willebrand factor | P04275 | VWF | ENSG00000110799 |
|  | YjeF N-terminal domain-containing protein 3 | A6XGL0 | YJEFN3 | ENSG00000250067 |
|  | Zinc finger C3H1 domain-containing protein | O60293 | ZFC3H1 | ENSG00000133858 |
|  | Zinc finger protein 57 homolog | Q9NU63 | ZFP57 | ENSG00000204644 |
|  | Zinc finger protein ZFPM2 | Q8WW38 | ZFPM2 | ENSG00000169946 |
|  | Zinc finger protein 551 | Q7Z340 | ZNF551 | ENSG00000204519 |
|  | Zinc finger SWIM domain-containing protein 6 | Q9HCJ5 | ZSWIM6 | ENSG00000130449 |
|  | Immunoglobulin lambda constant 3 | P0CG06 | IGLC3 | ENSG00000211679 |
|  | Hemoglobin subunit alpha | P69905 | HBA1 | ENSG00000206172 |
|  | Golgi-associated RAB2 interactor protein 4 | Q8IYT1 | GARIN4 | ENSG00000162771 |

**Supplementary Table 2** The 119 overlapping differentially expressed genes in the aptamer-based serum proteome and tissue transcriptome of gastric cancer

| **No** | **Gene name** | **Gene ID** | **Protein names** | **Protein ID** |
| --- | --- | --- | --- | --- |
|  | HBA1 | ENSG00000206172 | Hemoglobin subunit alpha | P69905 |
|  | APOE | ENSG00000130203 | Apolipoprotein E | P02649 |
|  | LYZ | ENSG00000090382 | Lysozyme C | P61626 |
|  | C1QC | ENSG00000159189 | Complement C1q subcomponent subunit C | P02747 |
|  | ASS1 | ENSG00000130707 | Argininosuccinate synthase | P00966 |
|  | ECHS1 | ENSG00000127884 | Enoyl-CoA hydratase | P30084 |
|  | SERPINA1 | ENSG00000197249 | Alpha-1-antitrypsin | P01009 |
|  | SAA1 | ENSG00000173432 | Serum amyloid A-1 protein | P0DJI8 |
|  | ILF2 | ENSG00000143621 | Interleukin enhancer-binding factor 2 | Q12905 |
|  | TRIM28 | ENSG00000130726 | Transcription intermediary factor 1-beta | Q13263 |
|  | RAB25 | ENSG00000132698 | Ras-related protein Rab-25 | P57735 |
|  | JCHAIN | ENSG00000132465 | Immunoglobulin J chain | P01591 |
|  | FN1 | ENSG00000115414 | Fibronectin | P02751 |
|  | GPI | ENSG00000105220 | Glucose-6-phosphate isomerase | P06744 |
|  | CD14 | ENSG00000170458 | Monocyte differentiation antigen CD14 | P08571 |
|  | FLNA | ENSG00000196924 | Filamin-A | P21333 |
|  | SPTAN1 | ENSG00000197694 | Spectrin alpha chain, non-erythrocytic 1 | Q13813 |
|  | CCDC47 | ENSG00000108588 | PAT complex subunit CCDC47 | Q96A33 |
|  | C3 | ENSG00000125730 | Complement C3 | P01024 |
|  | APOL1 | ENSG00000100342 | Apolipoprotein L1 | O14791 |
|  | THBS1 | ENSG00000137801 | Thrombospondin-1 | P07996 |
|  | ITGAV | ENSG00000138448 | Integrin alpha-V | P06756 |
|  | CKAP2 | ENSG00000136108 | Cytoskeleton-associated protein 2 | Q8WWK9 |
|  | PAPSS2 | ENSG00000198682 | Bifunctional 3'-phosphoadenosine 5'-phosphosulfate synthase 2 | O95340 |
|  | HUWE1 | ENSG00000086758 | E3 ubiquitin-protein ligase HUWE1 | Q7Z6Z7 |
|  | CPD | ENSG00000108582 | Carboxypeptidase D | O75976 |
|  | PRKCD | ENSG00000163932 | Protein kinase C delta type | Q05655 |
|  | ROCK2 | ENSG00000134318 | Rho-associated protein kinase 2 | O75116 |
|  | GSN | ENSG00000148180 | Gelsolin | P06396 |
|  | GALE | ENSG00000117308 | UDP-glucose 4-epimerase | Q14376 |
|  | DSN1 | ENSG00000149636 | Kinetochore-associated protein DSN1 homolog | Q9H410 |
|  | SHROOM3 | ENSG00000138771 | Protein Shroom3 | Q8TF72 |
|  | CLPX | ENSG00000166855 | ATP-dependent Clp protease ATP-binding subunit clpX-like | O76031 |
|  | KIF22 | ENSG00000079616 | Kinesin-like protein KIF22 | Q14807 |
|  | CHD1 | ENSG00000153922 | Chromodomain-helicase-DNA-binding protein 1 | O14646 |
|  | IGSF8 | ENSG00000162729 | Immunoglobulin superfamily member 8 | Q969P0 |
|  | OFD1 | ENSG00000046651 | Centriole and centriolar satellite protein OFD1 | O75665 |
|  | VWF | ENSG00000110799 | von Willebrand factor | P04275 |
|  | MCM5 | ENSG00000100297 | DNA replication licensing factor MCM5 | P33992 |
|  | RELCH | ENSG00000134444 | RAB11-binding protein RELCH | Q9P260 |
|  | GFM2 | ENSG00000164347 | Ribosome-releasing factor 2 | Q969S9 |
|  | FERMT3 | ENSG00000149781 | Fermitin family homolog 3 | Q86UX7 |
|  | KRT10 | ENSG00000186395 | Keratin, type I cytoskeletal 10 | P13645 |
|  | TRPM4 | ENSG00000130529 | Transient receptor potential cation channel subfamily M member 4 | Q8TD43 |
|  | VPS13C | ENSG00000129003 | Intermembrane lipid transfer protein VPS13C | Q709C8 |
|  | DUSP6 | ENSG00000139318 | Dual specificity protein phosphatase 6 | Q16828 |
|  | DEDD | ENSG00000158796 | Death effector domain-containing protein | O75618 |
|  | RDH14 | ENSG00000240857 | Retinol dehydrogenase 14 | Q9HBH5 |
|  | CHD7 | ENSG00000171316 | Chromodomain-helicase-DNA-binding protein 7 | Q9P2D1 |
|  | ZFC3H1 | ENSG00000133858 | Zinc finger C3H1 domain-containing protein | O60293 |
|  | CLU | ENSG00000120885 | Clusterin | P10909 |
|  | GGA1 | ENSG00000100083 | ADP-ribosylation factor-binding protein GGA1 | Q9UJY5 |
|  | TRMT1 | ENSG00000104907 | tRNA -N)-dimethyltransferase -N) methyltransferase) G26)dimethyltransferase) | Q9NXH9 |
|  | TUFT1 | ENSG00000143367 | Tuftelin | Q9NNX1 |
|  | SELPLG | ENSG00000110876 | P-selectin glycoprotein ligand 1 | Q14242 |
|  | ELMO2 | ENSG00000062598 | Engulfment and cell motility protein 2 | Q96JJ3 |
|  | GAB2 | ENSG00000033327 | GRB2-associated-binding protein 2 | Q9UQC2 |
|  | MACF1 | ENSG00000127603 | Microtubule-actin cross-linking factor 1, isoforms 1/2/3/4/5 | Q9UPN3 |
|  | TRIM13 | ENSG00000204977 | E3 ubiquitin-protein ligase TRIM13 | O60858 |
|  | SAA2 | ENSG00000134339 | Serum amyloid A-2 protein | P0DJI9 |
|  | INPP5F | ENSG00000198825 | Phosphatidylinositide phosphatase SAC2 | Q9Y2H2 |
|  | PGM2L1 | ENSG00000165434 | Glucose 1,6-bisphosphate synthase | Q6PCE3 |
|  | ACYP1 | ENSG00000119640 | Acylphosphatase-1 | P07311 |
|  | TNRC6B | ENSG00000100354 | Trinucleotide repeat-containing gene 6B protein | Q9UPQ9 |
|  | MDN1 | ENSG00000112159 | Midasin | Q9NU22 |
|  | KCNH2 | ENSG00000055118 | Potassium voltage-gated channel subfamily H member 2 | Q12809 |
|  | CAPRIN2 | ENSG00000110888 | Caprin-2 | Q6IMN6 |
|  | ZNF551 | ENSG00000204519 | Zinc finger protein 551 | Q7Z340 |
|  | PIN4 | ENSG00000102309 | Peptidyl-prolyl cis-trans isomerase NIMA-interacting 4 | Q9Y237 |
|  | TMEM39B | ENSG00000121775 | Transmembrane protein 39B | Q9GZU3 |
|  | NOCT | ENSG00000151014 | Nocturnin | Q9UK39 |
|  | ARMC8 | ENSG00000114098 | Armadillo repeat-containing protein 8 | Q8IUR7 |
|  | C4BPA | ENSG00000123838 | C4b-binding protein alpha chain | P04003 |
|  | NSUN4 | ENSG00000117481 | 5-methylcytosine rRNA methyltransferase NSUN4 | Q96CB9 |
|  | ALDH5A1 | ENSG00000112294 | Succinate-semialdehyde dehydrogenase -dependent succinic semialdehyde dehydrogenase) | P51649 |
|  | HBB | ENSG00000244734 | Hemoglobin subunit beta | P68871 |
|  | ACTG2 | ENSG00000163017 | Actin, gamma-enteric smooth muscle | P63267 |
|  | BNIP1 | ENSG00000113734 | Vesicle transport protein SEC20 | Q12981 |
|  | MAP3K9 | ENSG00000006432 | Mitogen-activated protein kinase kinase kinase 9 | P80192 |
|  | RPAP2 | ENSG00000122484 | Putative RNA polymerase II subunit B1 CTD phosphatase RPAP2 | Q8IXW5 |
|  | CROCC | ENSG00000058453 | Rootletin | Q5TZA2 |
|  | RTTN | ENSG00000176225 | Rotatin | Q86VV8 |
|  | SETD6 | ENSG00000103037 | N-lysine methyltransferase SETD6 | Q8TBK2 |
|  | ALPI | ENSG00000163295 | Intestinal-type alkaline phosphatase | P09923 |
|  | ANKHD1 | ENSG00000131503 | Ankyrin repeat and KH domain-containing protein 1 | Q8IWZ3 |
|  | TCF21 | ENSG00000118526 | Transcription factor 21 | O43680 |
|  | CFI | ENSG00000205403 | Complement factor I | P05156 |
|  | IGFBP2 | ENSG00000115457 | Insulin-like growth factor-binding protein 2 | P18065 |
|  | SARS2 | ENSG00000104835 | Serine--tRNA ligase synthetase) | Q9NP81 |
|  | C4A | ENSG00000244731 | Complement C4-A | P0C0L4 |
|  | MSH5 | ENSG00000204410 | MutS protein homolog 5 | O43196 |
|  | H-13 | ENSG00000124575 | Histone H1.3 | P16402 |
|  | APOB | ENSG00000084674 | Apolipoprotein B-100 | P04114 |
|  | ITIH2 | ENSG00000151655 | Inter-alpha-trypsin inhibitor heavy chain H2 | P19823 |
|  | PNMA2 | ENSG00000240694 | Paraneoplastic antigen Ma2 | Q9UL42 |
|  | APOA1 | ENSG00000118137 | Apolipoprotein A-I | P02647 |
|  | DSG1 | ENSG00000134760 | Desmoglein-1 | Q02413 |
|  | CP | ENSG00000047457 | Ceruloplasmin | P00450 |
|  | BVES | ENSG00000112276 | Blood vessel epicardial substance | Q8NE79 |
|  | ZFPM2 | ENSG00000169946 | Zinc finger protein ZFPM2 | Q8WW38 |
|  | YJEFN3 | ENSG00000250067 | YjeF N-terminal domain-containing protein 3 | A6XGL0 |
|  | LTF | ENSG00000012223 | Lactotransferrin | P02788 |
|  | FCN3 | ENSG00000142748 | Ficolin-3 | O75636 |
|  | TRPM5 | ENSG00000070985 | Transient receptor potential cation channel subfamily M member 5 | Q9NZQ8 |
|  | C7 | ENSG00000112936 | Complement component C7 | P10643 |
|  | AKAP6 | ENSG00000151320 | A-kinase anchor protein 6 | Q13023 |
|  | AMBP | ENSG00000106927 | Protein AMBP | P02760 |
|  | GPR17 | ENSG00000144230 | Uracil nucleotide/cysteinyl leukotriene receptor | Q13304 |
|  | TREH | ENSG00000118094 | Trehalase | O43280 |
|  | LBP | ENSG00000129988 | Lipopolysaccharide-binding protein | P18428 |
|  | MASP1 | ENSG00000127241 | Mannan-binding lectin serine protease 1 | P48740 |
|  | KRT5 | ENSG00000186081 | Keratin, type II cytoskeletal 5 | P13647 |
|  | F2 | ENSG00000180210 | Prothrombin | P00734 |
|  | KRT2 | ENSG00000172867 | Keratin, type II cytoskeletal 2 epidermal | P35908 |
|  | APOA4 | ENSG00000110244 | Apolipoprotein A-IV | P06727 |
|  | APOC3 | ENSG00000110245 | Apolipoprotein C-III | P02656 |
|  | KRT1 | ENSG00000167768 | Keratin, type II cytoskeletal 1 | P04264 |
|  | KRT4 | ENSG00000170477 | Keratin, type II cytoskeletal 4 | P19013 |
|  | KRT6C | ENSG00000170465 | Keratin, type II cytoskeletal 6C | P48668 |

**Supplementary Table S3** Baseline clinical data of the included patients

| Characteristics | Gastric cancer (n = 30) | Normal control (n = 22) | P value |
| --- | --- | --- | --- |
| Gender, n (%) |  |  | 0.01 |
| Male | 20 (74.1) | 7 (25.9) |  |
| Female | 10 (40.0) | 15 (60.0) |  |
| Age, median (IQR) | 66.5 (58.8, 71) | 34 (25.3, 44.8) | < 0.001 |
| WBC (×10^9^/L), mean ± SD | 5.0 ± 1.4 | 5.7 ± 1.3 | 0.076 |
| RBC (×10^12^/L), median (IQR) | 4.0 (3.6, 4.3) | 4.5 (4.4, 4.9) | < 0.001 |
| HGB (g/L), mean ± SD | 115.3 ± 21.9 | 140.8 ± 13.3 | < 0.001 |
| PLT (×10^9^/L), median (IQR) | 208 (165.8, 229.8) | 212.5 (181, 245.3) | 0.677 |
| AFP (ng/mL), median (IQR) | 2.1 (1.6, 3.1) | 2.0 (1.7, 3.3) | 1.000 |
| CEA (ng/mL), median (IQR) | 2.3 (1.6, 3.4) | 1.4 (0.9, 2.0) | 0.003 |
| CA199 (U/mL), median (IQR) | 10.5 (7.4, 13.4) | 9.4 (6.7, 13.2) | 0.756 |
| CA125 (U/mL), median (IQR) | 8.1 (6.5, 12.8) | 13.0 (8.6, 20.5) | 0.094 |
| ILF2 serum level (ng/ml), mean ± SD | 443.2 ± 303.3 | 72.3 ± 45.2 | < 0.001 |

IQR, Interquartile Range; SD: Standard Deviation.


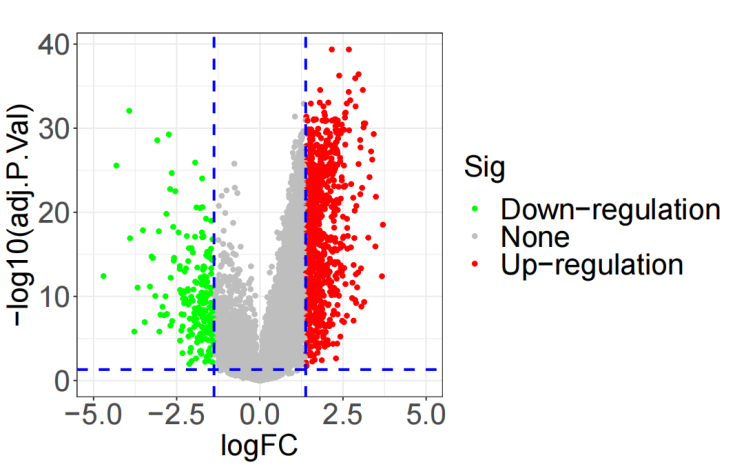


**Supplementary Figure 1** Volcano plot of differentially expressed genes in the transcriptome of gastric cancer tissues in the TCGA-STAD dataset.


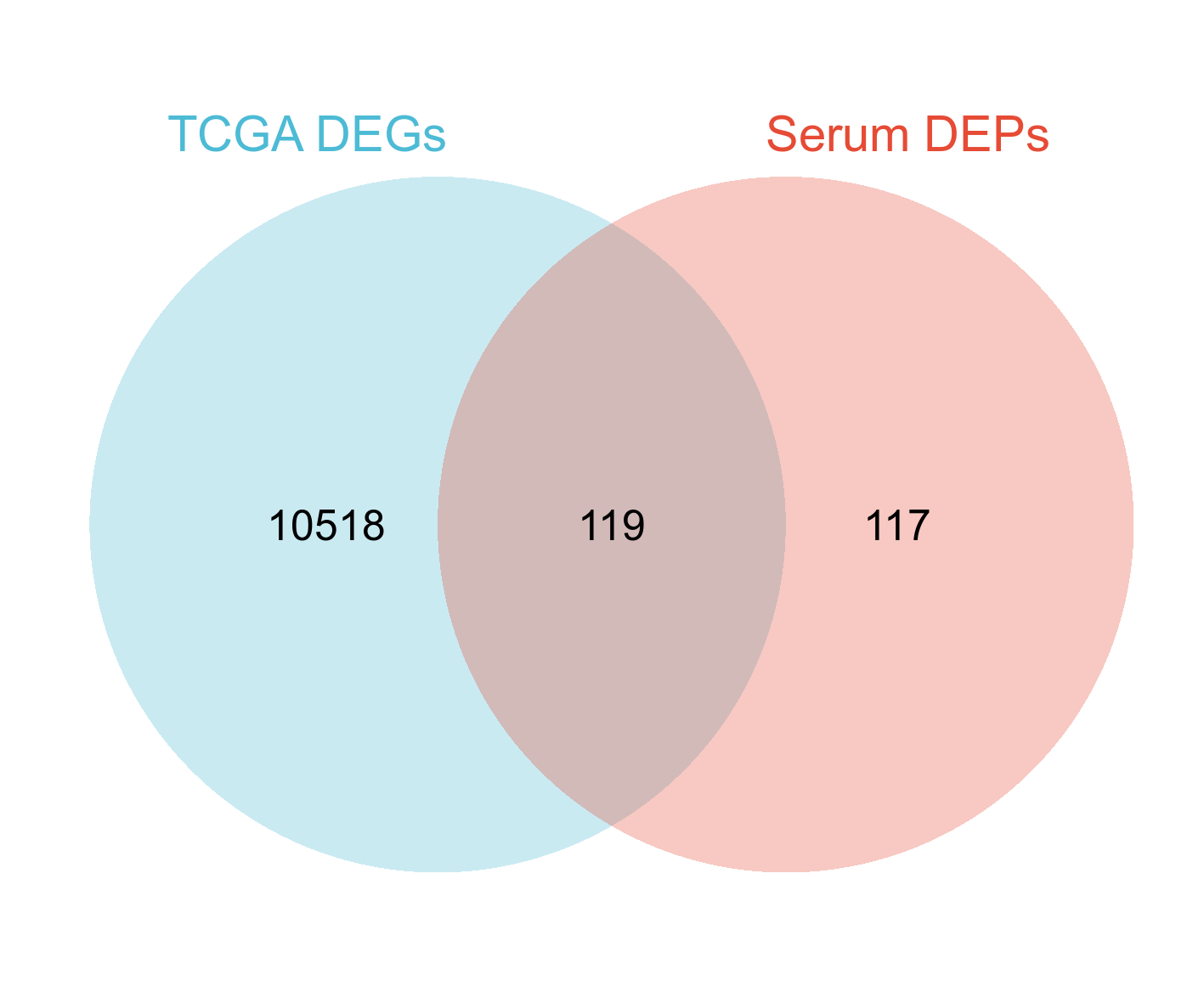


**Supplementary Figure 2** Venn diagram of the overlap of genes corresponding to differentially expressed proteins (DEPs) in the gastric cancer serum proteome and differentially expressed genes (DEGs) in the gastric cancer tissue transcriptome.


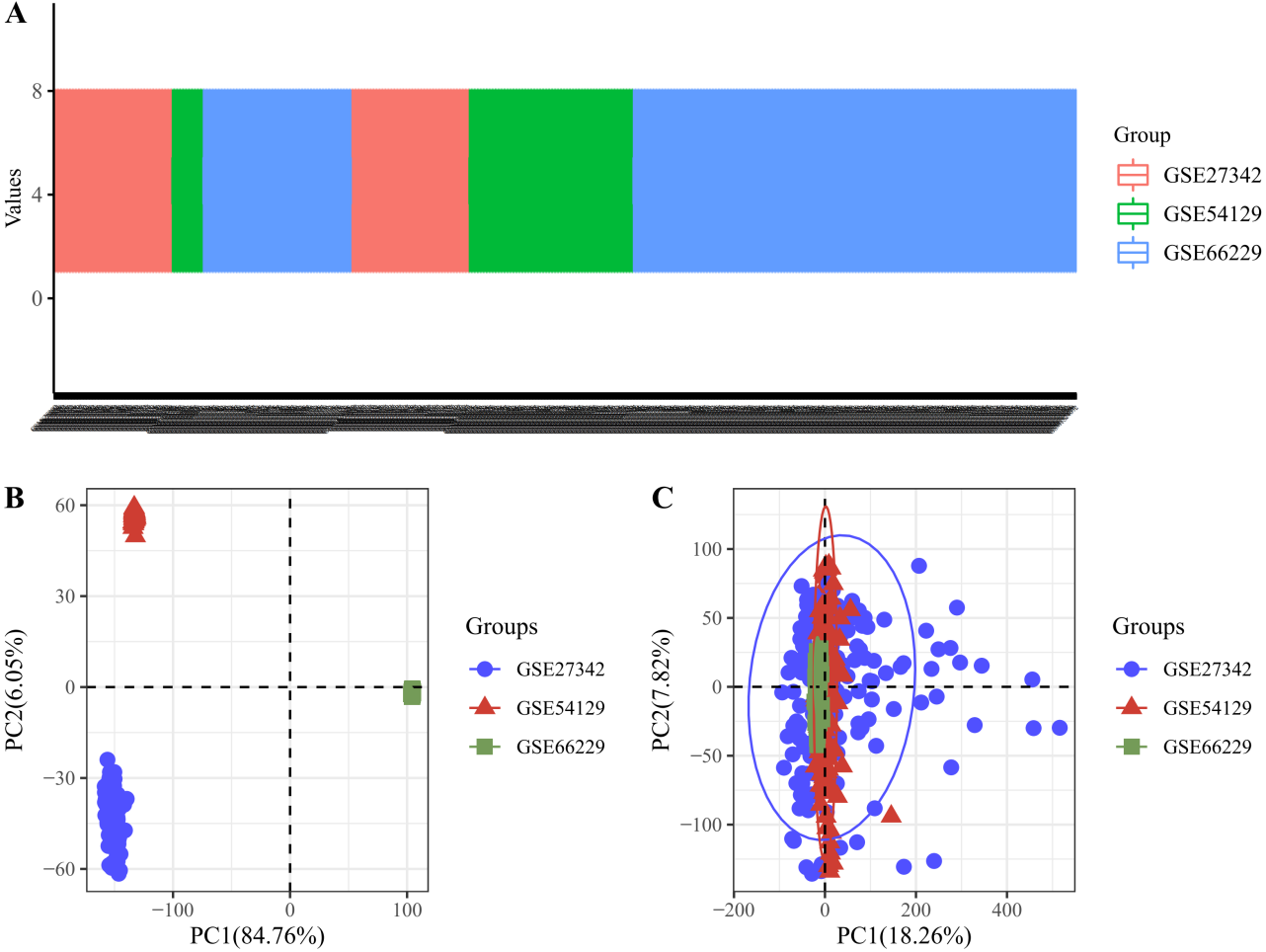


**Supplementary Figure 3** Batch effect correction in the three Gene Expression Omnibus (GEO) datasets. **A**: Box plots after data standardization, with different colors representing different datasets, rows representing samples, and columns representing gene expression values within samples; **B, C**: Principal component analysis (PCA) results before and after batch removal, with different colors representing different datasets.
